# Recurrent Sterile Pleuropericarditis After Withdrawal of Renal Transplant Immunosuppression in Anuric ESRD: A Case of Suspected IL‐1–Mediated Autoinflammation

**DOI:** 10.1155/crrh/2469859

**Published:** 2026-08-20

**Authors:** Evelien van Gelderen, Phillip Wagner, Ross Gilbert

**Affiliations:** ^1^ Department of Medicine, Johns Hopkins University School of Medicine, Baltimore, Maryland, USA, jhu.edu; ^2^ Department of Medicine, Division of Hospital Medicine, Johns Hopkins University School of Medicine, Baltimore, Maryland, USA, jhu.edu; ^3^ Department of Medicine, Division of Rheumatology, Johns Hopkins University School of Medicine, Baltimore, Maryland, USA, jhu.edu

## Abstract

Recurrent pericarditis and serositis pose a significant diagnostic challenge in patients with end‐stage renal disease (ESRD) and a history of extensive immunosuppression. A 29‐year‐old man with anuric ESRD secondary to IgA nephropathy on intermittent hemodialysis, with a history of 2 failed kidney transplantations followed by posttransplant nephrectomies and recent withdrawal of immunosuppression, presented with recurrent fevers, pericarditis, pleural effusions, and pericardial effusions. Fevers were unresponsive to multiple courses of broad‐spectrum antibiotics. A broad malignancy workup, including imaging, PET scan, cytology, and microbial cell‐free DNA was unremarkable. Multiple rounds of blood, pleural fluid, and pericardial fluid studies were negative for infectious etiologies, and serologic studies were unremarkable. Given the negative workup and clinical response to anti‐inflammatory therapy, his presentation was most consistent with an IL‐1–mediated autoinflammatory phenotype such as idiopathic recurrent pericarditis or undifferentiated systemic autoinflammatory disorder. Rilonacept was attempted as an IL‐1–targeted alternative because of long‐term colchicine toxicity concerns in ESRD; however, exposure was limited, and sustained disease control occurred after resumption of colchicine. This case highlights the need for further study of IL‐1 inhibitor use in ESRD patients, a population with limited representation in landmark recurrent pericarditis trials such as RHAPSODY and AIRTRIP.

## 1. Introduction

Recurrent pericarditis and pleural effusions in a patient with end stage renal disease (ESRD) and a history of immunosuppression are complex to diagnose and manage. The differential diagnosis is broad and includes infectious, malignant, autoimmune, and autoinflammatory conditions [1]. In this case presentation, we describe a patient with ESRD secondary to IgA nephropathy with a history of kidney transplantation who was previously on immunosuppressive agents. This patient has since had recurrent admissions for chest pain and dyspnea and was found to have pericarditis and pleural effusions. Malignant and infectious causes were ruled out. His case highlights the difficulty of diagnosing autoinflammatory syndromes as well as the challenge of treating patients with concomitant ESRD and serositis.

## 2. Case Report

**FIGURE 1:**
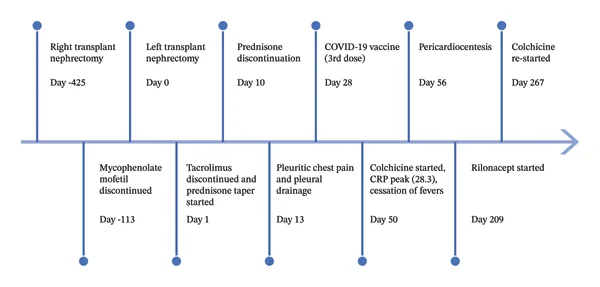
Timeline of notable events.

A 29‐year‐old man with anuric ESRD secondary to IgA nephropathy now on intermittent hemodialysis presented with recurrent fevers, pericarditis, pleural effusions, and pericardial effusions. His episodes of pericarditis were characterized by pleuritic chest pain and dyspnea. He was initially diagnosed with IgA nephropathy in 2009, and his disease progressed to anuric ESRD requiring kidney transplantation in 2013 and again in 2021. Both grafts failed secondary to biopsy‐proven antibody‐mediated rejection despite treatment with steroids, intravenous immunoglobulin (IVIG), tacrolimus, and mycophenolate mofetil. To protect PHI, we will refer to the day of his second nephrectomy as Day 0. He underwent bilateral transplant nephrectomies: the first on Day 425 (right) and the second on Day 0 (left). In terms of his immunosuppressive regimen, mycophenolate mofetil was discontinued on Day 113 and tacrolimus was discontinued on Day 1 (Figure 1). His immediate postoperative course was further complicated by acute hypoxemic respiratory failure thought to result from flash pulmonary edema or community‐acquired pneumonia (CAP) with negative cultures. His family history was notable for recurrent pancreatitis in his father and paternal grandmother, without any known rheumatologic or autoimmune disease.

On Day 9, he presented to the emergency department (ED) with acute‐onset right‐sided chest pain and dyspnea. Chest CT angiography demonstrated bilateral pleural effusions, ground‐glass opacities, and evidence of volume overload. He was on prednisone 5 mg which was tapered and discontinued on Day 10. He also completed a 7‐day empiric course of antibiotics for CAP with negative cultures. Shortly afterward, he re‐presented with right‐sided pleuritic chest pain and abdominal pain. He had leukocytosis and improved pleural effusions compared to the prior admission, but new radiographic opacities concerning for a new pneumonia. A broad infectious workup was again negative, including bacterial, yeast, mycobacterial, and fungal blood cultures; CMV, EBV, beta‐D‐glucan, galactomannan, and a respiratory pathogen panel were also negative. CT imaging showed a moderate right pleural effusion. Our infectious disease team was concerned about a parapneumonic effusion with possible empyema. Interventional pulmonology then performed thoracentesis on Day 13. Studies showed WBC 7413 (neutrophilic predominance), LDH 394 (serum LDH 234), Protein 4.4 (serum protein 6.3), Glucose 119, and pH 7.34, consistent with an exudative effusion (Table 1). Cultures and cytology studies were unrevealing. In consultation with transplant infectious disease, he was initially on vancomycin, cefepime, and metronidazole, later deescalated to cefepime monotherapy. On discharge, he was transitioned to cefpodoxime for a 14‐day course.

**TABLE 1:** Select laboratory tests upon admission.

Test (units)	Result (normal range)
Hgb (g/dL)	9.1 (12.6–17.4)
WBC (K/cu mm)	15.8 (4.5–11.0)
ESR (mm/hr)	104 (1–15)
CRP (mg/dL)	15.6 (≤ 0.5)
Eosinophil absolute (K/cu mm)	0.4 (0.12–0.30)
Albumin (g/dL)	3.9 (3.5–5.3)
Total protein (g/dL)	6.3 (6.0–8.2)
Lactate dehydrogenase (U/L)	234 (118–273)
IgA (mg/dL)	435 (61–348)
ANA	1:160 nucleolar pattern (negative)
Anti‐dsDNA	Negative (negative)
Anti‐Smith (immunofluorescence units)	Negative (negative)
Anticardiolipin (immunofluorescence units)	Negative (negative)
Anti‐beta‐2‐glycoprotein (immunofluorescence units)	WNL (WNL)
DRVVT	Negative (negative)
Anti‐RNP (immunofluorescence units)	Negative (negative)
Ro (immunofluorescence units)	Negative (negative)
La (immunofluorescence units)	Negative (negative)
ANCA	Negative (negative)
Ferritin (ng/mL)	535 (25–336)
Pleural fluid studies:	
WBC count (/cu mm)	7413 (0–499)
RBC count (/cu mm)	3000 (0–99)
Neutrophils (%)	79 (0–24)
Albumin (g/dL)	2.6
Glucose (mg/dL)	119
LDH (U/L)	394
pH	7.34
Protein (g/dL)	4.4
Pericardial fluid studies:	
WBC count (/cu mm)	2365 (0–499)
RBC count (/cu mm)	171,000 (0–99)
Neutrophils (%)	41 (0–24)
Protein (g/dL)	4.6
Glucose (mg/dL)	71
Troponin (0, 1, 3 h) April 9th	13, 12, 10 (≤ 20 ng/L)
Troponin (0, 1, 3 h) April 13th	8, 8, 8 (≤ 20 ng/L)
Troponin (0, 3 h) May 12th	15, 14 (≤ 20 ng/L)

*Note:* The patient completed GenomeSeqDx/clinical genome sequence analysis, mitochondrial disorders/sequence analysis and deletion testing of the mitochondrial genome; MicroarrayDx: whole genome chromosomal microarray.

Following receipt of a COVID‐19 vaccine on Day 28, he again developed dyspnea and chest pain as well as daily fevers. CT of chest, abdomen, and pelvis showed bilateral consolidations and a right‐sided pleural effusion with pleural thickening and enhancement. A chest tube was placed, draining 800 mL of fluid; pleural studies again demonstrated an exudative effusion. Broad infectious testing was again negative, and he was treated empirically with broad‐spectrum antibiotics due to perceived clinical trajectory.

While hospitalized, he experienced near‐daily fevers up to 39°C despite treatment with multiple broad‐spectrum antibiotics, including piperacillin–tazobactam, vancomycin, and subsequently meropenem. Microbial cell‐free DNA testing and repeated infectious disease evaluations were unrevealing. At this point in the hospitalization, the multidisciplinary assessment favored a noninfectious inflammatory process. Quantitative immunoglobulin testing showed a normal IgG level (743 mg/dL; reference 610–1616). PET imaging demonstrated diffuse FDG‐avid lymphadenopathy, although no lymph nodes were safely accessible for biopsy. Other findings were nonspecific and included moderate bilateral pleural effusions with thickening in the pleura with mild FDG avidity and small pericardial effusion. Serial imaging also showed pericarditis with an enlarging pericardial effusion for which he ultimately required pericardiocentesis given tamponade physiology. Echocardiography on Day 56 showed low‐normal left ventricular (LV) systolic function with an ejection fraction of 50%–55%, normal LV wall thickness and wall motion, normal right ventricular (RV) systolic function, no aortic valve stenosis, structurally normal mitral valve with trace mitral valve regurgitation, normal tricuspid valve with trace tricuspid valve regurgitation, a plethoric IVC with decreased respirophasic variation, and RV diastolic impingement and RA systolic impingement consistent with tamponade. There was a moderate‐to‐large‐sized pericardial effusion (2.0 cm posterior to the LV and 1.1 cm anterior to the RV in the parasternal long axis view). A postpericardiocentesis TTE showed small residual pericardial effusion around the right atrium and moderate‐to‐severe tricuspid regurgitation. Pericardial fluid studies were inflammatory and hemorrhagic (2365 WBCs, 170,000 RBCs, 41% neutrophils, 4.6 g/dL protein, 71 mg/dL glucose, and no LDH obtained). The pericardial fluid samples had no growth of bacteria, fungi, or mycobacteria and had unremarkable cytology. Given recurrent pericardial effusions, colchicine and ibuprofen were initiated, after which his fevers resolved and his serositis stabilized.

Rheumatology consultants were following and considered both autoimmune and autoinflammatory diseases. SLE was considered given the presence of serositis and positive ANA, but the patient did not meet 2012 SLICC classification criteria, as all disease‐specific autoantibodies were negative and complement levels were normal. Systemic sclerosis was considered given the nucleolar ANA pattern but was excluded by the absence of expected clinical findings. ANCA‐associated vasculitis was excluded by negative ANCA, MPO, and PR3 serologies and due to the absence of hallmark findings. Eosinophilic granulomatosis with polyangiitis was excluded based on several findings: the absolute eosinophil count of 400/μL did not meet the threshold for eosinophilia (≥ 500/μL), ANCA was negative, no eosinophils were identified in pleural or pericardial fluid analysis, and the patient lacked chronic rhinosinusitis, asthma, or cutaneous manifestations. IgG4‐related disease was considered but deemed unlikely as IgG subclass testing was unremarkable, and the clinical presentation was not consistent. While the family history of recurrent pancreatitis (father and paternal grandmother) could raise consideration for familial IgG4‐related autoimmune pancreatitis, the father’s heavy alcohol use and hypertriglyceridemia provide more plausible explanations for his pancreatic disease. FMF was considered given the patient’s Lebanese ancestry, but the patient’s age of onset, fever duration, and lack of rash pointed away from FMF. Whole genome sequencing revealed no pathogenic MEFV variants; TRAPS was also considered but excluded by the absence of TNFRSF1A variants. CAPS was excluded based on negative NLRP3 testing; MKD was excluded by the absence of expected findings with negative MVK testing. Adult‐onset Still’s disease was considered given high spiking fevers and elevated ferritin, but the patient lacked characteristic features like a salmon‐colored rash or arthritis. The ferritin elevation was modest and nonspecific in the context of acute inflammation and ESRD.

This patient underwent comprehensive whole‐genome sequencing rather than a targeted autoinflammatory gene panel. No pathogenic or likely pathogenic variants were identified. Whole genome chromosomal microarray and mitochondrial DNA sequencing were also performed and were negative, with only two mitochondrial variants of uncertain significance identified (MT‐ND5, MT‐TT).

Since these hospitalizations, his outpatient rheumatologist started rilonacept in place of colchicine in Month 6 due to the high risk of colchicine toxicity in ESRD. The patient stopped rilonacept in Month 8 after receiving 8 doses due to diffuse body aches, fatigue, nausea, and diarrhea temporally associated with starting the medication, although it was noted that he may also have had recurrent viral infections that could explain these symptoms. He was then switched back to colchicine but is under consideration for initiating anakinra. He continues to have recurrent presentations to the ED and admissions for chest pain and body pain, with the cause thought to be multifactorial including anxiety, chronic pain, and volume overload. While on colchicine, he has had no further episodes of serositis or fever, and his ESR/CRP levels have been closer to normal.

## 3. Discussion

This patient’s clinical picture of recurrent sterile serositis with high spiking fevers, markedly elevated acute phase reactants (CRP > 15 mg/dL and ESR > 100 mm/hr), a negative infectious workup, and a dramatic response to colchicine is most consistent with an autoinflammatory disease driven by dysregulated innate immunity.

The rationale for IL‐1 blockade is based on both the clinical phenotype and the pathophysiology of idiopathic recurrent pericarditis. In recurrent pericarditis, initial pericardial injury triggers inflammasome activation in pericardial macrophages and mesothelial cells [1, 2]. In susceptible individuals, this leads to a self‐perpetuating cycle of IL‐1β‐mediated inflammation and recurrent flares. Colchicine’s efficacy in this setting is mediated in part through inhibition of NLRP3 inflammasome assembly and IL‐1β release. This patient’s rapid defervescence within 24 h of colchicine initiation, with concurrent normalization of inflammatory markers and stabilization of effusions, strongly supported an IL‐1‐driven process and provided the mechanistic rationale for pursuing IL‐1‐targeted therapy.

Alternative etiologies were considered and deemed less likely. The extensive infectious evaluation has been negative. Previously diffuse lymphadenopathy is regressing, arguing against malignancy. Uremic pericarditis is considered less likely given adherence to dialysis (peak BUN of 46 mg/dL). Notably, fevers and serositis did not correlate with dialysis adequacy and were unresponsive to antibiotics, yet resolved promptly with colchicine. The COVID‐19 vaccine may have served as an immune trigger in a patient with a primed innate immune system following withdrawal of immunosuppression, though causality remains uncertain. Recurrent sterile serositis, fevers, elevated inflammatory markers, colchicine responsiveness, and a possible flare after an immune stimulus point to an IL‐1‐mediated disease process.

The diagnostic label that best captures this presentation is uSAID, which encompasses a heterogeneous category of patients with clinical features of systemic autoinflammation who do not meet criteria for a defined syndrome and lack an identifiable pathogenic variant [3, 4]. The core features are consistent: unprovoked recurrent episodes of systemic inflammation, fever, serositis, elevated acute phase reactants during flares with normalization between them, and the absence of high‐titer autoantibodies. Miao et al. have highlighted that uSAID is an expanding diagnostic category, with many patients eventually receiving more precise diagnoses as novel variants, and pathways are characterized [3]. Idiopathic recurrent pericarditis and uSAID likely represent overlapping phenotypes along a shared autoinflammatory spectrum rather than distinct entities. Both feature episodic sterile inflammation, flare‐associated inflammatory marker elevation, the absence of pathogenic autoantibodies, and response to innate immune‐targeted therapy [4, 5]. What matters clinically is that recognizing that this overlap supports the rationale for IL‐1 blockade and should prompt clinicians to consider a broader autoinflammatory differential when evaluating patients with recurrent serositis of unclear etiology.

The management of idiopathic recurrent pericarditis and uSAID follows a similar framework. NSAIDs and colchicine are both used first, with escalation to biologic therapy in refractory or high‐risk cases [4]. In this patient, colchicine achieved disease control, but the cumulative toxicity risk in the setting of ESRD made long‐term continuation a concern. This prompted consideration for targeted IL‐1 blockade.

The AIRTRIP trial established anakinra, a recombinant IL‐1 receptor antagonist, as an effective option for colchicine‐resistant recurrent pericarditis, demonstrating meaningful reductions in recurrence [6]. RHAPSODY followed, supporting rilonacept, a soluble decoy receptor targeting both IL‐1α and IL‐1β, as a well‐tolerated alternative with more convenient dosing [7]. One important gap across both trials is the underrepresentation of patients with advanced CKD and ESRD, which complicates direct application to this case. Radin et al. addressed this specifically, showing that hemodialysis did not meaningfully alter rilonacept pharmacokinetics and that no dose adjustment may be required in ESRD. The study was limited, however, as the population included 6 patients, and only single‐dose pharmacokinetics was studied [8]. These limitations are such that the updated Food and Drug Administration (FDA) label for rilonacept notes that no studies have reliably investigated the pharmacokinetics of rilonacept in those with renal failure [9]. More data on efficacy, safety, and optimal duration of rilonacept and other IL‐1 blocking medications in patients with ESRD are needed.

This case highlights the diagnostic challenges of distinguishing idiopathic recurrent pericarditis from undifferentiated systemic autoinflammatory disease and underscores the need for ongoing reassessment as new clinical and genetic insights emerge.

## Funding

The authors did not receive financial support for this manuscript.

## Ethics Statement

Written informed consent was obtained from the patient, and the patient’s privacy is protected through anonymization.

## Conflicts of Interest

The authors declare no conflicts of interest.

## Data Availability

Data sharing is not applicable to this article as no datasets were generated or analyzed during the current study.
